# Detection of serum anti-candidalysin IgG by indirect ELISA: a novel auxiliary tool for diagnosing invasive candidiasis in a preliminary pediatric study

**DOI:** 10.1128/spectrum.03245-24

**Published:** 2025-08-05

**Authors:** Ting Luo, Xun Li, Haipeng Yan, Ling Gong, Longlong Xie, Xiangyu Wang, Jiaotian Huang, Yufan Yang, Xiao Li, Yingying Zhang, Guoping Lu, Zhenghui Xiao, Xiulan Lu

**Affiliations:** 1 Pediatrics Research Institute of the Affiliated Children’s Hospital of Xiangya School of Medicine, Central South University (Hunan Children’s Hospital) 12570, Changsha, China; 2 Department of Pediatric Intensive Care Unit & Hunan Provincial Key Laboratory of Emergency Medicine for Children, Hunan Children's Hospital 37046, Changsha, China; 3 University of South China Hengyang Medical School 159373, Hengyang, China; 4 Department of Pediatric Intensive Care Unit, Children's Hospital of Fudan University, National Children's Medical Center 145601, Shanghai, China; 5 Shanghai Institute of Infectious Disease and Biosecurity, Fudan University 12478, Shanghai, China; Naturwissenschaftliches und Medizinisches Institut an der Universitat Tubingen, Reutlingen, Germany

**Keywords:** invasive candidiasis, diagnosis, immunoserology, biomarker, *Candida albicans*

## Abstract

Invasive candidiasis (IC) is the most common fungal infection in clinical settings, yet early diagnosis remains challenging. Candidalysin, a key virulence factor in invasive infections, exhibits site-specific pathogenicity. Our study identifies candidalysin as a serodominant antigenic peptide and reveals significantly elevated serum anti-candidalysin IgG levels in IC across diverse anatomical sites, suggesting its potential as a novel diagnostic biomarker. To evaluate this, we developed an indirect enzyme-linked immunosorbent assay (ELISA) to quantify serum anti-candidalysin IgG. The assay was validated in two distinct validation subsets using a 5-year cohort comprising 121 proven, probable, or possible IC cases from Hunan Children’s Hospital, alongside 105 non-IC controls (patients with other infections and healthy individuals). In validation set 1, comparing proven IC cases (*n* = 20) to non-IC controls, the cutoff increased to 0.1768 ng/mL, with an area under the curve (AUC) of 0.818 (95% CI: 0.736–0.899), 80% sensitivity, and 73.3% specificity. In validation set 2, receiver operating characteristic (ROC) analysis comparing proven/probable IC cases (*n* = 77) to non-IC controls (*n* = 105) yielded an AUC of 0.870 (95% CI: 0.821–0.919) at an optimal cutoff of 0.1647 ng/mL, demonstrating 87% sensitivity and 70.5% specificity. These findings establish the indirect ELISA for anti-candidalysin IgG as a robust diagnostic tool for IC. Additionally, the assay effectively discriminates colonization from invasive infection at non-sterile sites in suspected cases, offering valuable guidance for antifungal therapy and prophylaxis.

**IMPORTANCE:**

The indirect enzyme-linked immunosorbent assay (ELISA) for detecting serum anti-candidalysin IgG exhibits robust diagnostic performance in invasive candidiasis, demonstrating particular utility in distinguishing commensal colonization from invasive infection in cases of suspected deep-seated candidiasis. This assay represents a valuable adjunctive tool for both clinical diagnosis and therapeutic management of invasive candidiasis.

## INTRODUCTION

*Candida albicans* is a significant human opportunistic fungal pathogen, commonly found as a harmless commensal organism on the skin, gastrointestinal tract, oral cavity, and vulvovaginal tract in up to 75% of healthy individuals (1). However, in immunocompromised and critically ill patients, it can lead to invasive candidiasis (IC), a life-threatening systemic infection, with an estimated attributable mortality rate of 65% (2).

IC consists of candidemia and deep-seated candidiasis. Three situations that need to be considered when diagnosing invasive candidiasis: (i) candidemia in the absence of deep-seated candidiasis; (ii) candidemia associated with deep-seated candidiasis; and (iii) deep-seated candidiasis that is not associated with candidemia (3). Currently, there is no precise method for diagnosing invasive candidiasis. While blood culture remains the gold standard for detecting candidemia, its sensitivity is suboptimal, ranging from 21% to 71% (4), and its processing time can take 2–5 days. Moreover, blood culture is not suitable for diagnosing deep-seated candidiasis. For such infections, tissue histopathology from normally sterile sites is considered the gold standard; however, this method requires invasive biopsy sampling, which is often not practical in clinical settings and is infrequently used.

Additionally, the most widely employed non-culture diagnostic method (1, 3)-β-D-glucan detection (BDG assay) is associated with a high false-positive rate. This is due to interference from factors such as surgical gauze, albumin or globulin infusions, hemodialysis, and certain polysaccharide-based antineoplastic drugs. Although various diagnostic approaches are available for IC, none provide sufficiently sensitive detection of *Candida*. Autopsy studies have revealed that approximately 38% of patients with *Candida* identified post-mortem had negative blood cultures pre-mortem, suggesting that nearly half of such cases may go undiagnosed (3, 5).

Furthermore, *Candida albicans*, a common commensal organism in humans, does not necessarily indicate an invasive infection when detected. Differentiating between colonization and invasive infection remains a significant challenge in the diagnosis of IC (6, 7). In intensive care units, where respiratory support devices are frequently used, sputum cultures often yield high rates of *Candida* detection, as *C. albicans* is a frequent colonizer of the oral and respiratory tracts. This complicates the interpretation of positive results, making it difficult to determine whether they indicate infection. Consequently, the decision to treat patients with *Candida* isolated from the respiratory tract remains a subject of ongoing debate (8).

The hyphal form of *Candida albicans* is known to contribute to its pathogenicity during invasive candidiasis (9, 10). Candidalysin, a highly expressed hyphal-specific extracellular peptide of *C. albicans*, has been identified as a critical pathogenic factor in *C. albicans* infections (11).

In this study, we found that candidalysin is a serodominant antigen in patients with *Candida* infections. Furthermore, we developed an indirect enzyme-linked immunosorbent assay (ELISA) to detect serum anti-candidalysin IgGs. Our findings demonstrate that candidalysin antibodies hold potential as a biomarker for invasive candidiasis, and the detection of anti-candidalysin IgG could represent a novel diagnostic approach for *Candida albicans* infections.

## MATERIALS AND METHODS

### Sera collection and study population

The sera samples from 121 patients diagnosed with invasive candidiasis or suspected cases at Hunan Children’s Hospital have been collected between January 2019 and June 2024. The recruitment criterion of the cohort is that the patients with positive *Candida* cultures or positive BDG assays are all included; positive cultures could derive from different clinical specimens of body fluid, such as blood, sputum, stool, urine, ascites, bile, and cerebrospinal fluid. All patients whose serum collection time to their positive culture time was over 14 days should be excluded. The study population was classified into four groups: proven IC (candidemia and deep-seated candidiasis, *n* = 20), probable IC (*n* = 57), possible IC (*n* = 44), and non-IC groups (*n* = 105). The invasive *Candida albicans* infection diagnostic criteria are basically according to the consensus of IFD from EORTC and MSGERC in 2019, with some modifications (12). Proven IC is defined as a positive culture from a normally sterile site of the host. Probable IC includes immunocompromised patients with positive *Candida albicans* urine, stool, or sputum cultures at least twice, as well as a host factor and a clinical feature. Possible IC includes patients with positive BDG assays at least twice. The non-IC group consists of other fungal infections, bacterial infections, viral infections, and healthy controls. Healthy controls were pediatric individuals without any basic disease or acute infection. The workflow of the study is illustrated in Fig. 3 in detail. The ELISA method was validated using two distinct cohort subsets: validation set 1 compared proven IC cases (*n* = 20) against non-IC controls (*n* = 105) to establish true positive identification, while validation set 2 employed an expanded population including both proven and probable IC cases (*n* = 77) to determine true negative detection rate and help screen out risk samples in a large population. The study design for the development and validation of this ELISA method is schematically illustrated in Fig. 3. Demographic information and clinical test results were extracted from electronic medical records and statistically analyzed in Table 1 . All serum samples were collected using sterile, pyrogen-free, and additive-free containers and stored at −80°C until use. The study protocol was approved by the Medical Ethics Committee of Hunan Children’s Hospital (approval no. HCHLL-2022-80).

**TABLE 1:** Demographic and clinical characteristics of IC group and non-IC groups^*a*^

Variable	IC group	Other infections	Healthy control	P_IC_	P_IC_
	*n* = 77	*n* = 47	*n* = 57	vs other inf.	vs healthy ctrl
Age, month	13 (3.3, 62)	7.5 (3.4, 23.5)	36 (12, 72)	**0.0377**	0.871
Sex					
Male	50 (64.9)	36 (76.6)	40 (70.2)	0.172	0.523
Female	27 (35.1)	11 (23.4)	17 (29.8)	0.172	0.523
Diesease background					
Solid tumor	1 (1.3）	0^*c*^	/^*b*^	0.316	NA^*d*^
Malignancy	10 (13.0）	0	/	**0.001**	NA
Shock	9 (11.7）	0	/	**0.002**	NA
Organ dysfunction					
Respiratory	41 (53.2)	18 (38.3)	/	**0.0001**	NA
Stomach/intestine	33 (42.9)	3 (6.4)	/	**1.12E-08**	NA
Brain	21 (27.3)	10 (21.3)	/	0.0271	NA
Kidney	19 (24.7)	0	/	**3.23E-06**	NA
Heart	21 (27.3)	2 (4.3)	/	**1.74E-05**	NA
Treatment					
Antibiotics	44 (57.1)	30 (63.8)	/	0.461	NA
Immunosuppressive therapy	8 (10.4)	0	/	**0.0036**	NA
Outcomes					
Discharge	62 (80.5)	45 (95.7)	/	**0.0029**	NA
Death	11 (15.9)	2 (4.3)	/	**0.0091**	NA

^*a*^ Values were presented as *n* (%) or median (quartile 1, quartile 3). Bold values were statistically significant (*P* < 0.05).

^*b*^ "/” means healthy controls do not have any symptoms of severe disease background and organ dysfunction.

^*c*^ 0 means there are no evidence of specific mentioned disease background or organ dysfunction.

^*d*^ NA, not applicable.

### Dot blot and qualitative reactivity assessment

Spot 1 ug candidalysin samples (1 µL) onto a dry polyvinylidene fluoride membrane alongside beta-glucan as positive controls. Block with 4% non-fat milk in TBST for 1 hour to prevent non-specific binding. Incubate with patients’ sera 1:1,000 diluted in TBST for 1–2 hours at room temperature. Wash 3× with TBST to remove unbound antibodies. Probe with HRP-conjugated secondary antibody for 1 hour at room temperature, then wash again. Develop signals using chromogenic substrates TMB for 30 minutes and stop the reaction with 2 M H_2_SO_4_. Detect signals at 450 nm by a microplate reader. In this study, a visible gray spot was defined as positive. Higher grayscale values indicate stronger signals. Beta-glucan was taken as a positive control.

### Optimization of indirect ELISA

Optimal working concentrations of ELISA reagents—including coating antigen, serum samples, secondary antibody, and blocking solution—were determined using checkerboard titration. The optimal antigen concentration and serum dilution were selected based on the highest optical density (OD450) ratio of positive to negative samples (P/N ratio). The indirect ELISA assay was finally set as follows. Candidalysin peptide was chemically synthesized by LifeTein. Ninety-six-well ELISA plates (Corning 96 Well EIA/RIA Plate, REF 3590) were coated with 1 µg of candidalysin peptide antigen per well in 100 µL of bicarbonate buffer (pH 9.6) and incubated overnight at 4°C. The plates were blocked with 200 µL of skim milk (3% wt/vol) in PBS containing 0.05% Tween-20 (PBST) for 3 hours at room temperature. After discarding the blocking buffer, the plates were washed twice with PBST. Serum samples (1:500 dilution or 1:250 dilution) along with positive standard controls were added for IgG or IgM detection, respectively, and incubated for 1 hour at room temperature. The plates were washed four times with PBST and then incubated with 100 µL of various dilutions of secondary antibody for 1 hour at room temperature to detect IgG and IgM against candidalysin. Following four additional washes with PBST, TMB substrate solution (Thermo Scientific Pierce 1-Step Ultra TMB ELISA substrate solution) was added for a chromogenic reaction lasting 5 minutes in the dark at room temperature. The reaction was stopped by adding 100 µL of 2 M sulfuric acid to each well, and the OD450 was measured using a BioTeK Synergy H1 microplate reader.

### Repeatability of indirect ELISA

The repeatability of the ELISA was evaluated. Intra-assay validation involved testing 10 positive and 10 negative serum samples in duplicates. For inter-assay variation, three serum samples were tested across 20 microplates, which represent positive samples with high anti-candidalysin IgG level, low level, and negative control samples, respectively. The mean, standard deviation (SD), and coefficient of variation (CV) for individual ELISAs were calculated.

### Data analysis

Serum anti-candidalysin IgG level is calculated by interpolating ODs to the standard curve of anti-candidalysin monoclonal mouse IgG. Serum anti-candidalysin IgM level is a relative value, which was expressed as the sample-to-positive (S/P) ratio, calculated as follows: (OD sample − OD negative control)/(OD positive control − OD negative control). The cutoff values for IgG and IgM detection were determined using receiver operating characteristic (ROC) curve analysis in IBM SPSS Statistics 22. The area under the ROC curve (AUC) measures diagnostic accuracy. In order to evaluate the diagnostic accuracy and reliability of this ELISA method, we conducted a method consistency assessment by comparing it with two other conventional methods, namely the BDG assay and microbiological cultures. The Cohen’s kappa coefficient (κ) assessed the agreement of the developed ELISA method with the BDG assay and microbiological culture, respectively. Differences between positive and negative groups in each ELISA were analyzed using the unpaired *t*-test in GraphPad Prism 6.01 software, with a *P* value of <0.05 considered significant. The inter-assay and intra-assay CVs were determined as the ratio of the SD to the mean OD450 value for each sample group.

### Correlation analysis between clinical indicators and serum anti-candidalysin IgG+IgM levels

In order to identify anti-candidalysin IgG-associated clinical indices, we have collected the data of routine blood tests, liver and kidney function, and coagulation functions, which were examined by Mindray hematology workflow CAL7000, Autobio automatic biochemical Analyzer FX8, and STAGO automatic coagulation Analyzer STAGO-R, respectively, in our clinical laboratory department. The involved clinical indices were listed in Table S3. Spearman’s rank correlation coefficient (ρ) was calculated to measure correlations between serum anti-candidalysin IgG+IgM levels and clinical indices. A two-tailed *P* value <0.05 was considered statistically significant. Data were visualized using scatter plots to illustrate the relationships.

## RESULTS

### Detection of anti-candidalysin IgG in serum samples as a potential biomarker of IC

**Fig 1:**
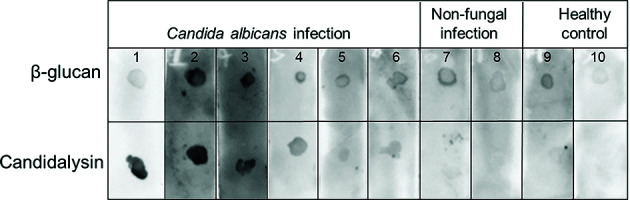
Dot blot for detection of serum anti-candidalysin IgG in the IC, non-fungal infection patients, and healthy control individuals, IC group: lane 1 and 2, serum samples from two candidemia patients with septic shock; lane 3 and 4, serum samples from two candidemia patients; lane 5 and 6, from two invasive candidiasis patients. Control groups: lane 7, serum sample from one bacterial sepsis patient; lane 8, from one viral sepsis; lane 9 and 10, from two healthy children.

Candidalysin, a hyphae-specific secreted peptide of *C. albicans*, plays a critical role in invasive candidiasis. To assess its antigenic potential, we screened serum samples from six patients with invasive candidiasis and four disease controls for anti-candidalysin IgG using dot blot assays. Candidalysin demonstrated more precise detection capabilities for *C. albicans* infections compared to a common pan-fungal biomarker, β-glucan (Fig. 1), and showed potential as a diagnostic biomarker for IC.

### Determination of the antigenic site of candidalysin

**Fig 2:**
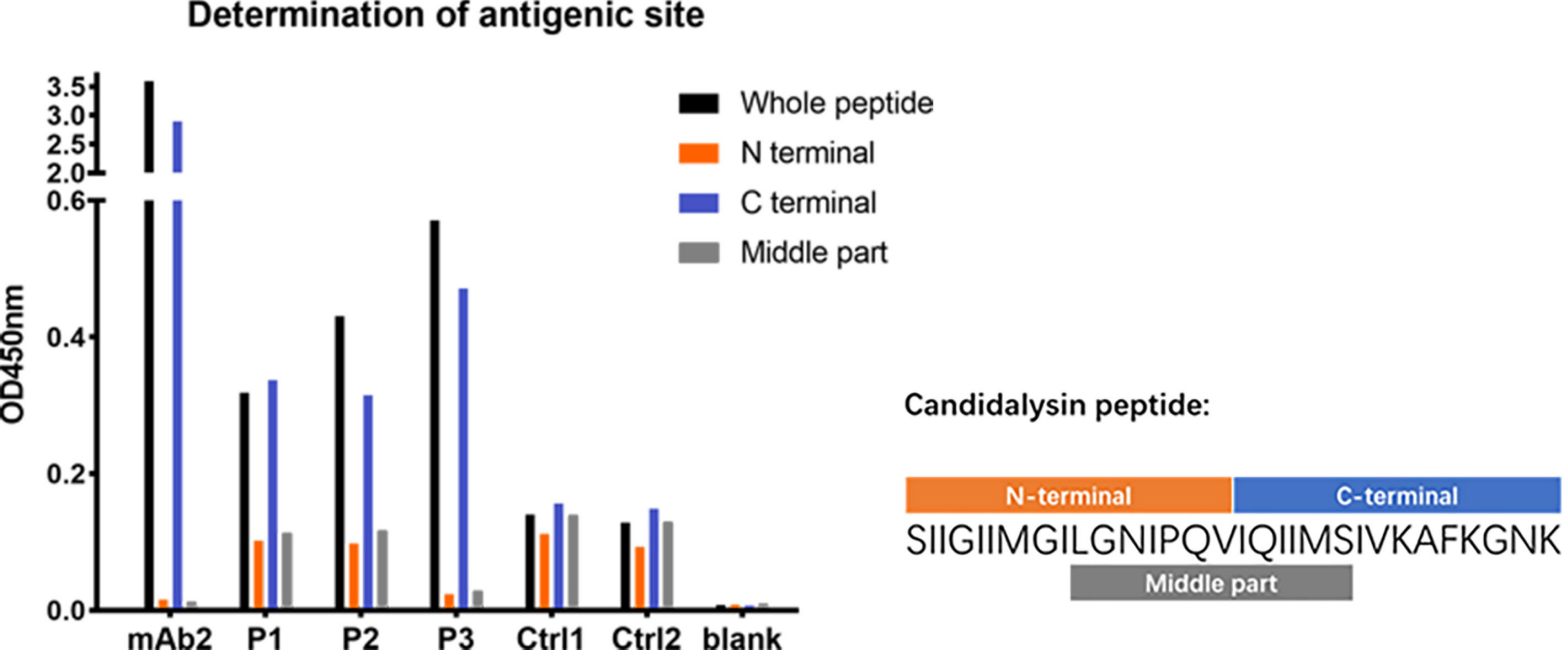
Determination of antigenic site of peptide candidalysin by ELISA. mAb2: mouse anti-candidalysin IgG monoclonal antibody#2; P1, P2, and P3 represent serum samples from three *Candida*-infected patients; Ctrl1 and Ctrl2 represent serum samples from two healthy individuals. Blank means ELISA tests without any sera addition.

To identify the major antigenic sites of candidalysin, we analyzed the serological responses to its N-terminal, C-terminal, and central regions. Results showed that anti-candidalysin IgG predominantly bound to the C-terminal region, particularly the last nine amino acids (IVKAFKGNK), which are rich in lysine. Mouse monoclonal antibodies also exhibited strong binding to this region, indicating it may be the primary antigenic site responsible for eliciting a host immune response (Fig. 2).

### Study design for development and validation of a novel diagnostic ELISA for *Candida albicans* infection

**Fig 3:**
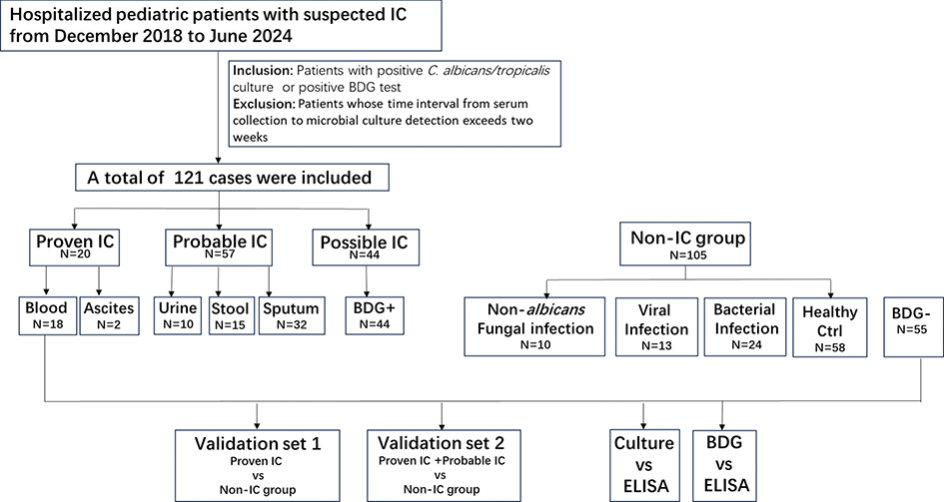
The flow chart of study design for development and validation of a novel diagnostic ELISA for *C. albicans* infection. The cohort of IC was comprised of patients with positive cultures for *C. albicans* or *Candida tropicalis* from any clinical specimen source, as well as patients with positive BDG assays at least twice.

To establish a novel diagnostic approach for *Candida albicans* infection, we developed and optimized an indirect ELISA method. The assay was subsequently validated in two distinct study populations to evaluate its diagnostic performance for invasive candidiasis. Validation set 1: compared proven IC cases (*n* = 20) against non-IC controls (*n* = 105) to determine true positive rates and particularly assess their diagnostic sensitivity. Validation set 2: included both proven and probable IC cases (*n* = 77) versus non-IC controls to establish true negative detection and help screening out risk samples in a broader clinical population, which evaluates the specificity of the diagnostic method. To further validate the reliability of this novel method, we conducted consistency testing with BDG assays and conventional fungal culture. A schematic representation of the study design is provided in Fig. 3.

### Construction and optimization of an indirect ELISA

To confirm the presence of candidalysin-specific antibodies in sera from *C. albicans*-positive patients, we developed an indirect ELISA to detect both anti-candidalysin IgG and IgM. Chemically synthesized candidalysin peptides served as the coating antigen, while a mouse anti-candidalysin IgG monoclonal antibody was used as the standard. We optimized parameters, including coating antigen concentration, blocking buffer composition, serum dilution, and secondary antibody dilution (Table S1 and Fig S1). The assay exhibited high repeatability, with intra-assay variation (CV < 10%) and inter-assay variation (CV < 20%) across multiple runs (Table S2).

### Validation of anti-candidalysin IgG as a diagnostic biomarker of invasive candidiasis

**Fig 4:**
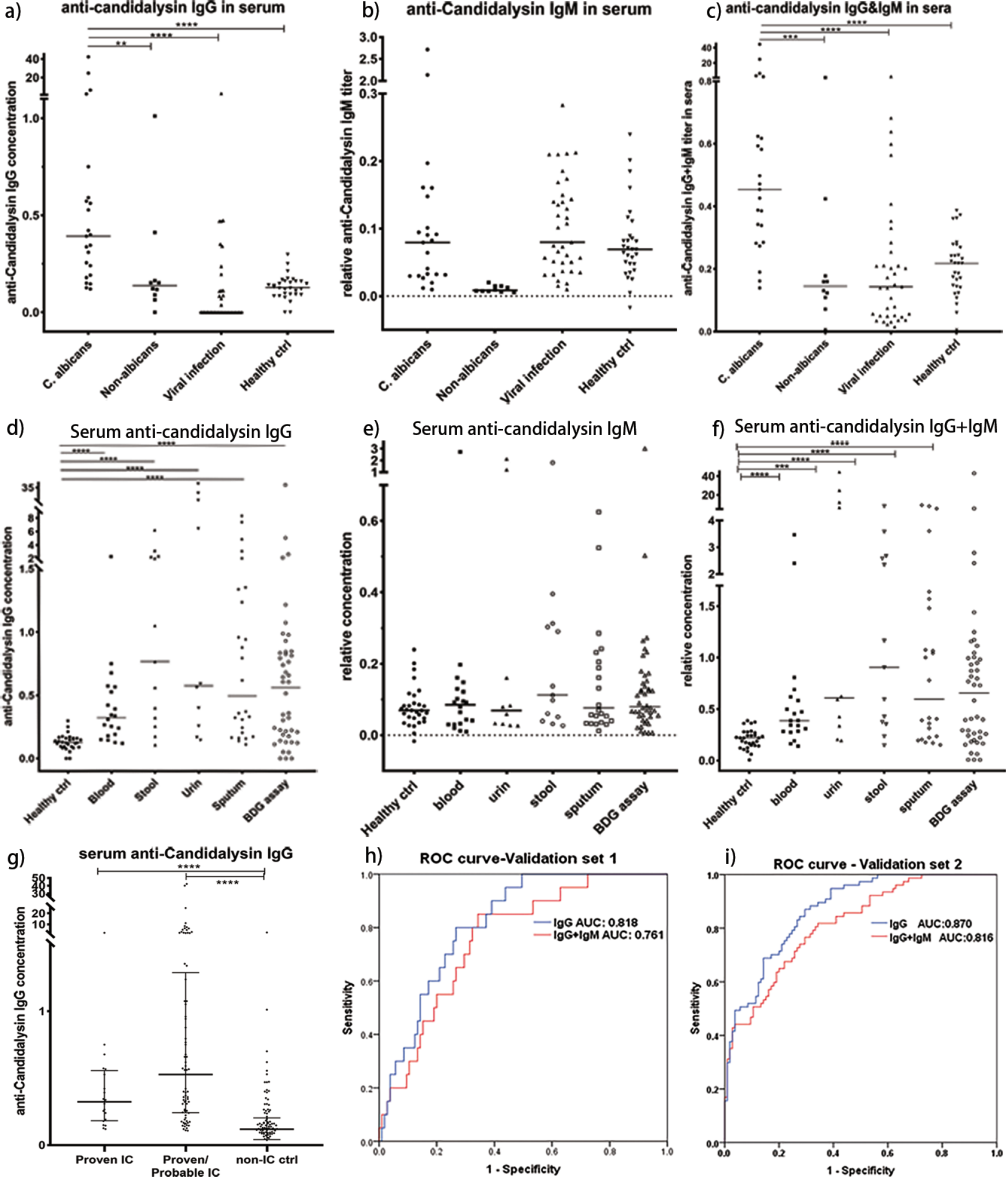
Detection of serum anti-candidalysin IgG or IgM in different patient groups based on our developed indirect ELISA assay. (**a–c**) Serum detection of anti-candidalysin IgG, IgM, and IgG+IgM antibody levels in validation set 1 (proven IC vs non-IC groups), respectively; (**d–f**) serum detection of anti-candidalysin IgG, IgM, or IgG+IgM antibody levels in validation set 2, respectively (IC groups vs non-IC groups, IC groups refer to all proven or probable IC cases); (**e**) comparison of anti-candidalysin IgG levels in proven IC, IC groups, and non-IC groups; (**h–i**) ROC analysis of anti-candidalysin IgG and anti-candidalysin IgG+IgM as diagnostic biomarker in validation sets 1 and 2, respectively.

The developed ELISA assay was subsequently validated in two distinct study populations to evaluate its diagnostic performance for IC. Validation set 1: comparing proven IC cases (*n* = 20) against non-IC controls (*n* = 105) revealed that anti-candidalysin IgG titers were significantly elevated in candidiasis patients (Fig. 4a). ROC analysis indicated that anti-candidalysin IgG is a promising diagnostic marker for *C. albicans* infections, with an AUC of 0.818 (95% CI: 0.736–0.899) at a cutoff of 0.1768 ng/mL, achieving sensitivities and specificities of 80.0% and 73.3% (Fig. 4h), respectively. IgM levels did not significantly differ between groups, likely due to variations in infection phases (Fig. 4b). However, combining IgG and IgM testing improved overall sensitivity to 85%, though it reduced specificity to 65.7% (Fig. 4c and h).

We further validated anti-candidalysin IgG as a biomarker in the validation set 2 of 77 positive IC samples and 105 non-IC control samples. The ELISA for anti-candidalysin IgG yielded an AUC of 0.870 (95% CI: 0.821–0.919), with sensitivities and specificities of 87.0% and 70.5%, respectively (Fig. 4g and i). Especially, we applied this method for evaluating deep-seated candidiasis at different infected sites, and it expanded our understanding of deep-seated candidiasis. In children with positive *C. albicans* cultures, the highest positivity rate of serum anti-candidalysin IgG assay was found in patients with positive stool cultures, followed by blood, urine, and sputum cultures, which indicates that serum anti-candidalysin IgG levels are also sensitive and responsive to deep-seated candidiasis (Table 2, Fig. 4d). Moreover, BDG-positive samples exhibited considerable variability in anti-candidalysin IgG levels, reflecting the distinct detection specificities of these assays (Fig. 4d–f).

**TABLE 2:** Positive rates in different infection sites based on the serum anti-candidalysin IgG ELISA assay

	Blood	Stool	Urine	Sputum	BDG assay
Pos (nr)	15	13	9	26	34
Total	18	15	10	32	44
Pos (%)	83.3	86.7	90	81.3	77.3
Median (ng/mL)	0.341	0.768	0.576	0.639	0.561

### Comparison of anti-candidalysin IgG ELISA with BDG assay and fungal culture

We evaluated the agreement between the anti-candidalysin IgG ELISA, the BDG assay, and fungal culture using kappa coefficients. The results demonstrated moderate agreement between the ELISA and fungal culture (κ = 0.555, *P* < 0.05), whereas only slight agreement was observed between the ELISA and the BDG assay (κ = 0.046, *P* < 0.05) (Table 3).

**TABLE 3:** Comparison of anti-candidalysin IgG indirect ELISA with fungal culture and BDG assay*^,^*

	ELISA for anti-candidalysin IgG	
	+	−
^*a*^Culture + (*n* = 77)	67^*c*^	10^*e*^
^*b*^Culture − (*n* = 105)	31^*f*^	74^*d*^
PPA^*g*^	87%	/^*i*^
NPA^*h*^	/^*i*^	70.5%
Kappa values	0.555	NA^*j*^

	ELISA for anti-candidalysin IgG	
	+	−
BDG assay + (*n* = 44)	39^*c*^	5^*e*^
BDG assay − (*n* = 55)	46^*f*^	9^*d*^
PPA^*g*^	88.6%	/^*i*^
NPA^*h*^	/^*i*^	16.4%
Kappa values	0.046	NA^*j*^

^*a*^ Culture positive indicates that fungal culture is *C. albicans* or *tropicals* positive.

^*b*^ Culture negative indicates that culture is *C. albicans* or *tropicals* negative, as well as all culture negative.

^*c*^ The number of true-positive samples.

^*d*^ The number of true-negative samples.

^*e*^ The number of false-negative samples.

^*f*^ The number of false-positive samples.

^*g*^ PPA: positive percent agreement, PPA =$\frac{\text{Positives in comparative method}}{\text{Positives in reference method}}\times 100\%=\frac{a}{a+c}\times 100\%$.

^*h*^ NPA: negative percent agreement, NPA=$\frac{\text{Negatives in comparative method}}{\text{Negatives in reference method}}\times 100\%=\frac{b}{b+d}\times 100\%$.

^*i*^ “/” means there is no result.

^*j*^ NA, Not applicable.

The ELISA presented strong positive percent agreement (PPA: 87%) with fungal culture, indicating that both methods exhibit comparable sensitivity in diagnosing IC. In contrast, comparison with the BDG assay revealed low negative percent agreement (NPA: 16.4%) but high positive percent agreement (PPA: 88.6%), suggesting that while the two assays have similar sensitivity, their specificity markedly differs.

This discrepancy is expected, as the BDG assay detects a pan-fungal marker, β-glucan, whereas the ELISA is specific to *C. albicans* and *Candida tropicalis* infections.

### Correlation of anti-candidalysin IgG levels with clinical indicators

**Fig 5:**
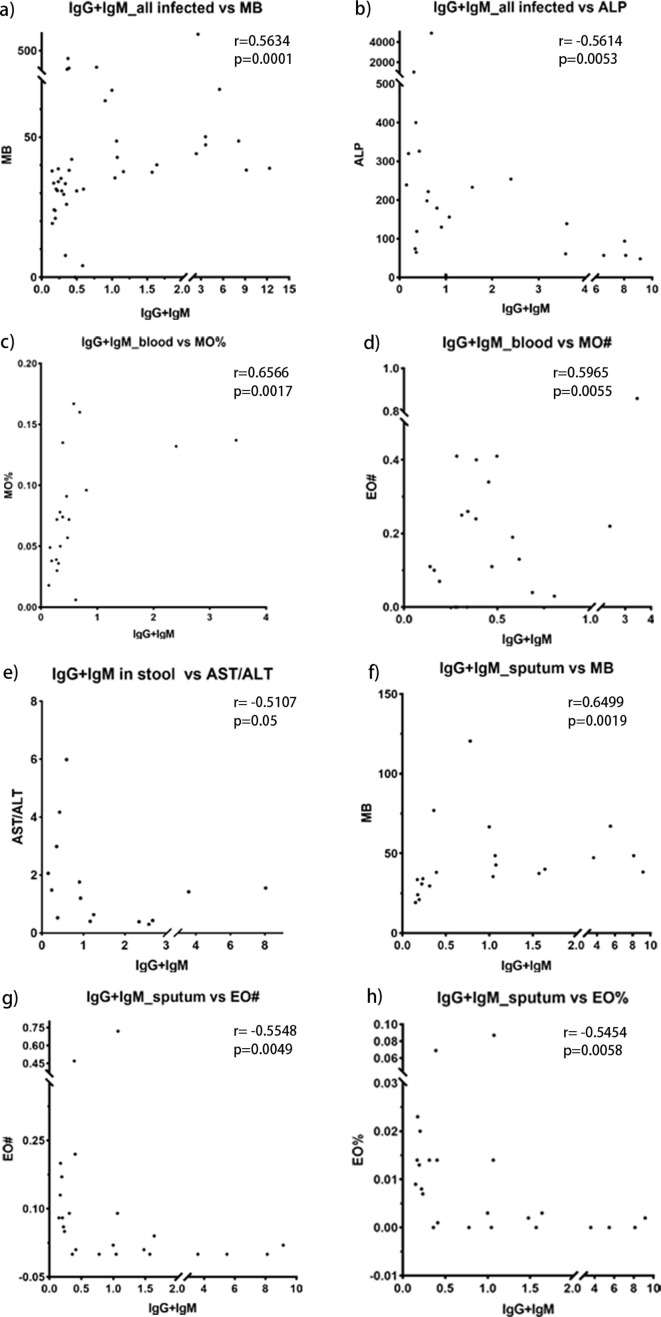
Correlation of serum anti-candidalysin IgG+IgM levels with clinical indicators. (a–b) Screening of sera samples from all the patients with positive *Candida* culture in any clinical specimen. (c–h) Screening of sera samples from the patients with positive *Candida* culture by blood (c–d), stool (e), or sputum (f–h) specimen, respectively. MB, myoglobin; ALP, alkaline phosphatase; AST/ALT, glutamic oxaloacetic transaminase/glutamic pyruvic transaminase; MO%, the ratio of mononuclear cells; MO#, mononuclear cell count; EO%, the ratio of eosinophils; EO#, eosinophil granulocyte count; BA#, basophil granulocyte count.

We explored the correlation between anti-candidalysin IgG levels and various clinical indicators. Generally, serum anti-candidalysin IgG and IgM levels correlated with serum myoglobin and alkaline phosphatase across all candidiasis patients (Fig. 5a and b). In candidemia cases, these antibodies correlated with mononuclear cell counts and percentage (Fig. 5c and d). In patients with stool cultures positive for *C. albicans*, IgG and IgM levels correlated with liver enzyme markers (aspartate aminotransferase [AST]/alanine aminotransferase [ALT]) (Fig. 5e). For sputum cultures, IgG and IgM levels correlated with myoglobin and eosinophil counts and percentage (Fig. 5f–h).

## DISCUSSION

### Serum anti-candidalysin IgG is a potential novel diagnostic biomarker of invasive candidiasis

Currently, there is a lack of precise and effective biomarkers for the diagnosis of invasive candidiasis (4). A major challenge is distinguishing between commensal colonization and invasive infection, as the host-pathogen interaction is prevalent in both states (13). Identifying serodiagnostic antigens from *C. albicans* that can differentiate these conditions with high sensitivity and specificity remains a complex task (14). In our study, candidalysin was identified as a serodominant antigen, with its C-terminal region serving as the principal immunogenic site. The performance of serum anti-Candidalysin IgG for the diagnosis of candidemia (validation set 1) and for the diagnosis of IC (validation set 2) is good, with AUC values of 0.818 and 0.870, respectively (Table 4). Interestingly, some samples from the healthy cohort exhibited anti-candidalysin IgG levels as high as those found in certain *Candida*-positive patients. This may be due, on the one hand, to some patients being in the very early phase of *Candida* infection, where specific IgG levels have begun to rise but are not yet markedly elevated. On the other hand, colonization in healthy individuals may not be limited to the yeast form of *C. albicans*. It is known that *C. albicans* morphotypes are strongly influenced by the local bacterial population and the host’s complex microenvironment. The hypha-specific peptide candidalysin can combat bacteria and support fungal commensalism in the gut (15). Consequently, *C. albicans* morphotype changes are highly dynamic, with multiple forms coexisting in the host. We propose categorizing colonization into two states: static and active. Whether *C. albicans* exists as a commensal colonizer or causes invasive infection depends largely on the host’s immune status. In immunosuppressed or critically ill patients, an active state of *Candida* can lead to the early stages of invasive infection. Therefore, when considered alongside the patient’s immune status, elevated anti-candidalysin IgG levels may serve as early indicators of invasive candidiasis, offering a valuable diagnostic tool for invasive candidiasis.

**TABLE 4:** List of diagnostic parameters of ELISA assays in this study^*c*^

Data set	Type	AUC	Accuracy (%)	Sensitivity (%)	Specificity (%)	FNR (%)	FPR (%)	PPV (%)	NPV (%)	Youden
IgG^*a*^	Validation set 1	0.818	74.4	80.0	73.3	26.7	20.0	80.0	73.3	0.533
IgG^*a*^+IgM^*b*^	Validation set 1	0.761	68.8	85.0	65.7	34.3	15.0	85.0	65.7	0.507
IgG^*a*^	Validation set 2	0.87	77.5	87.0	70.5	29.5	13.0	87.0	70.5	0.575
IgG^*a*^+IgM^*b*^	Validation set 2	0.816	72.5	81.8	65.7	34.3	18.2	81.8	65.7	0.475

^*a*^ Anti-candidalysin IgG in sera.

^*b*^ Anti-candidalysin IgM in sera.

^*c*^ FNR, false-negative rate; FPR, false-positive rate; NPV, negative predictive value; PPV, positive predictive value; Youden: Youden′s index = Sensitivity + Specificity − 1.

Furthermore, anti-candidalysin IgG in serum can differentiate infections caused by *C. albicans*, *C. tropicalis*, and *C. dubliniensis* from other *Candida* species and non-fungal infections (16). This species-specific diagnostic capability is instrumental in guiding antifungal treatment decisions.

### The indirect ELISA assay for detecting anti-candidalysin IgG represents a valuable auxiliary tool for diagnosing invasive candidiasis

Traditionally, microbiological culture and BDG assays are the routine clinical examinations for *Candida* infections (4, 17). However, the culture method is often insensitive to invasive candidiasis, while the BDG assay serves as a pan-diagnostic approach for fungal infections, lacking specificity (3, 17–22). Additionally, the BDG assay is susceptible to interference from various biological factors and treatments, leading to potential false positives (20–23). In contrast, our developed indirect ELISA for anti-candidalysin IgG detection demonstrates high sensitivity and specificity for *C. albicans* and *C. tropicalis* (Fig. 4h–i). This method aligns well with culture results but surpasses them in sensitivity, specificity, and turnaround time (Table 3). However, it does not correlate with BDG assay results, likely due to differences in the microbial spectrum detected by each method (Table 3). Notably, this ELISA method is particularly sensitive to invasive candidiasis across various infection sites. No matter the cultured positive results in stool, urine, or sputum, their serum samples all present positive rates above 80% by ELISA (Table 2), which facilitates the diagnosis of deep-seated candidiasis. In addition, our ELISA method utilizes serum samples, which reduces contamination concerns associated with other sample types, like stool, urine, and sputum. IgM is generally referred to as the acute infection phase. It usually has peaks of IgM level at 7–10 days after infection. However, our sample collection time is within 2 weeks before or after the microbiology culture report. The samples could be at an early acute phase or recovery phase, so that there is not a significant rise in IgM.

Despite its advantages, antibody testing may be affected in immunocompromised patients due to impaired antibody production resulting from immunosuppression. Interestingly, several studies have shown that antibody detection assays perform well in patients with neutropenia and cell-mediated immune defects (20, 24). In our study, approximately 15% of patients with proven or suspected IC exhibited serum globulin levels below the normal range. However, low total serum globulin did not appear to affect the anti-candidalysin IgG titer. Notably, one patient with the lowest globulin concentration had an extremely high anti-candidalysin IgG titer.

Nevertheless, the latest global guideline for the diagnosis and management of candidiasis (2025) has mentioned that molecular technique PCR is moderately recommended for any patient population or sample type in diagnosing invasive candidiasis, particularly in the absence of established alternative methods (e.g., matrix-assisted laser desorption ionization-time of flight mass spectrometry) or when rapid identification of potentially problematic species (e.g., *Candida auris*) is needed (25). Candida PCR has been focused on blood samples, with panfungal PCR and other broad-range PCR methods being generally used to test invasive samples (e.g., cerebrospinal fluid or tissue). Although PCR of *Candida* has high sensitivity and specificity, it is not always detected due to the limited number of *Candida* species. Besides, the detection of *Candida* DNA fragments does not help differentiate invasive hyphae from yeast, as *Candida* yeasts are commensals in humans.

### Anti-candidalysin IgG level correlates with clinical indicators depending on the infection body site

Since the discovery of candidalysin in 2016, numerous studies have highlighted its significant role across various host niches (11, 26–28). Understanding these site-specific characteristics is crucial for tailoring diagnostic and therapeutic strategies, as well as for improving patient outcomes.

In this study, the correlation of serum anti-candidalysin IgG and IgM levels with the clinical indices of the routine blood tests, liver and kidney function, and coagulation functions that are often impacted during fungal infections has been analyzed. It is found that high serum levels of anti-candidalysin IgG in patients with positive sputum samples for *Candida* have been associated with increased eosinophil percentage ratios and eosinophil granulocyte counts, which indicates allergic responses (Fig. 5g–i). It is reported that candidalysin, but not proteases, can promote allergic airway hyperresponsiveness by stimulating platelets through the von Willebrand factor receptor GP1bα and releasing the Wnt antagonist Dickkopf-1 (Dkk-1), which drives Th2 and Th17 cell responses essential for antifungal protection (29, 30). This pathogenic mechanism explained our clinical findings well. In the candidemia patients, the levels of anti-candidalysin IgG in serum positively correlate with the ratio of mononuclear cells and mononuclear cell count (Fig. 5c and d). Candidalysin can damage host monocyte membranes, providing an escape route from the hostile environment of phagosomes. Moreover, candidalysin induces NLRP3 inflammasome activation, which enhances the host-protective pro-inflammatory response in mononuclear phagocytes (31). Therefore, in candidemia, the interplay of candidalysin with monocytes plays an important role. In the patients with positive *Candida* in stool samples, the sera level of anti-candidalysin IgG is associated with the ratio of ALT and AST levels, indicating liver functionality (Fig. 5e). It has been reported that gut dysbiosis can induce intestinal inflammation and disrupt the gut barrier, allowing *C. albicans* exotoxin, candidalysin, to translocate to the liver and induce hepatocyte cytotoxicity (28). Furthermore, candidalysin has been linked to the severity and mortality of alcoholic hepatitis. These correlations are consistent with the documented mechanisms and indirectly underscore the high reliability of this ELISA assay.

### Conclusion and perspective

Overall, the indirect ELISA assay for detecting serum anti-candidalysin IgG represents higher sensitivity and specificity than fungal culture and β-D-glucan assays, respectively. Moreover, it helps in the discrimination of colonization and invasive infection. It could be a valuable auxiliary tool for diagnosing invasive candidiasis. However, it is limited to detecting candidalysin secreted by specific *Candida* species. It is recommended to incorporate additional pan-candida biomarkers, such as mannan, alongside anti-candidalysin IgG antibodies for a more comprehensive and precise diagnosis. Additionally, IgG titers alone cannot differentiate between the acute and recovery phases of infection. To improve this, the development of a standardized anti-candidalysin IgM monoclonal mouse antibody for ELISA assays is warranted. Lastly, it is important to note that all serum samples tested in this study were derived from pediatric patients. For broader applicability, validation of our assay in adult patient sera is essential. Expanding the sample size and conducting multicenter studies will be necessary to establish an accurate cutoff value for the diagnosis of invasive candidiasis.

## SUPPLEMENTAL MATERIAL

Supplemental material spectrum.03245-24-s0001.docx Tables S1 to S3; Fig S1.

ASM does not own the copyrights to Supplemental Material that may be linked to, or accessed through, an article. The authors have granted ASM a non-exclusive, world-wide license to publish the Supplemental Material files. Please contact the corresponding author directly for reuse.
