# Supplementary material for: Detection of serum anti-candidalysin IgG by indirect ELISA: a novel auxiliary tool for diagnosing invasive candidiasis in a preliminary pediatric study
Source: Microbiol Spectr. 2025 Aug 5;13(9):e03245-24. doi: 10.1128/spectrum.03245-24 (PMC12403709; doi:10.1128/spectrum.03245-24)
Supplement: Supplemental material — Tables S1 to S3; Fig S1. [file spectrum.03245-24-s0001.docx]

**Supplement**

**Table S1 Determination of optimal settings for ELISA assays of anti-candidalysin IgG and anti-candidalysin IgM.**

| ELISA | Protein antigen | Sample dilution | | Secondary antibody | | Blocking buffer | Diluent for sample and secondary antibody | |
| --- | --- | --- | --- | --- | --- | --- | --- | --- |
| IgG 10μg/ml | | 1:500 | 1:5000 | | | PBST in 3% skim milk | | PBST in 3% skim milk |
| IgM 10μg/ml | | 1:250 | 1:10000 | | PBST in 3% skim milk | | PBST in 3% skim milk | |

**Table S2. The repeatability of the developed ELISAs for intra-assay and inter-assay**

| **Analysis** | **Parameters** | **IgG detection** | **IgM detection** |
| --- | --- | --- | --- |
| **Intra-assay** | Range of SD | 0.002-0.103 | 0.001-0.049 |
|  | Average of SD | 0.018 | 0.018 |
|  | Range of CVs | 0.25-0.99% | 1.10-10.77% |
|  | Average of CVs | 3.76% | 3.85% |
|  |  |  |  |
| **Inter-assay** | S1: Mean ± 2SD | 0.691±0.278 | 0.105±0.025 |
|  | CVs of mean | 20.20% | 11.75% |
|  | S2: Mean ± 2SD | 0.084±0.027 | 0.153±0.029 |
|  | CVs of mean | 16.10% | 9.44% |
|  | S3: Mean ± 2SD | 0.085±0.029 | 0.084±0.014 |
|  | CVs of mean | 17.00% | 8.17% |

The repeatability of the developed ELISAs for intra-assay with three replicate tests of 10 positive and 10 negative samples and inter-assays with three replicates on 20 different plates of high positive, low positive and negative samples. SD: standard deviation; CV: coefficient of variation

**Table S3. List of Clinical Indices Associated with the Correlation between Serum Anti-Candidalysin IgGs and Clinical Parameters**

| **Tests** | **Clinical indices** |
| --- | --- |
| **Routine blood test** | White Blood Cell (WBC) |
|  | Red Blood Cell (RBC) |
|  | Hemoglobin (Hb) |
|  | Platelet (PLT) |
|  | Neutrophil Ratio (NE) |
|  | Lymphocyte Ratio (LY) |
|  | Monocyte Ratio (MO) |
|  | Eosinophil Ratio (EO) |
|  | Basophil Ratio (BA) |
|  | Neutrophil Count (NE#) |
|  | Lymphocyte Count (LY#) |
|  | Monocyte Count (MO#) |
|  | Eosinophil Count (EO#) |
|  | Basophil Count (BA#) |
|  | Hematocrit (HCT) |
|  | Mean Corpuscular Volume (MCV) |
|  | Mean Corpuscular Hemoglobin (MCH) |
|  | Mean Corpuscular Hemoglobin Concentration (MCHC) |
|  | Red Cell Distribution Width Coefficient of Variation (RDW-CV) |
|  | Plateletcrit (PCT) |
|  | Mean Platelet Volume (MPV) |
|  | Platelet Distribution Width (PDW) |
|  | Whole Blood C-Reactive Protein (Whole Blood CRP) |
|  |  |
| **Liver and kidney function** | Total Bilirubin (TBIL) |
|  | Direct Bilirubin (DBIL) |
|  | Indirect Bilirubin (IBIL) |
|  | Total Protein (TP) |
|  | Albumin (ALb) |
|  | Globulin (GLO) |
|  | Albumin/Globulin Ratio (A/G) |
|  | Alanine Transaminase (ALT) |
|  | Aspartate Aminotransferase (AST) |
|  | AST/ALT |
|  | Total Bile Acid (TBAC) |
|  | Alkaline Phosphatase (ALP) |
|  | Gamma-Glutamyl Transferase (GGT) |
|  | Urea |
|  | Creatinine (Cre) |
|  | Urea/Creatinine Ratio (Urea/Cre) |
|  | Uric Acid (UA) |
|  | Lactate Dehydrogenase (LD) |
|  | Creatine Kinase (CK) |
|  | Creatine Kinase-MB (CK-MB) |
|  | Myoglobin (MB) |
|  | Troponin T (TNIU) |
|  | N-terminal Pro-Brain Natriuretic Peptide (PRO-BNP) |
|  |  |
| **Coagulation functions** | Prothrombin Time (PT) |
|  | International Normalized Ratio (INR) |
|  | Activated Partial Thromboplastin Time (APTT) |
|  | Fibrinogen (FIB) |
|  | Thrombin Time (TT) |
|  | Antithrombin III (AT3) |





**Figure S1** **Determination of optimal settings for ELISA assays of anti-candidalysin IgG and anti-candidalysin IgM**

The optimal coating antigen amount, dilution of IgG antibody, IgM antibody and secondary antibody were tested using OD450 ratio between positive sample and negative control sample (S/N value). The highest S/N value was defined as the optimal concentration of the coated antigen of each ELISA.
